# Bacterial lipopolysaccharide is associated with stroke

**DOI:** 10.1038/s41598-021-86083-8

**Published:** 2021-03-22

**Authors:** Marisa Hakoupian, Eva Ferino, Glen C. Jickling, Hajar Amini, Boryana Stamova, Bradley P. Ander, Noor Alomar, Frank R. Sharp, Xinhua Zhan

**Affiliations:** 1 Department of Neurology, University of California Davis School of Medicine, Sacramento, CA USA; 2 Division of Neurology, Department of Medicine, University of Alberta, Edmonton, AB Canada; 3 Department of Neurology and MIND Institute, University of California Davis Medical Center, 2805 50th Street, Sacramento, CA 95817 USA

**Keywords:** Stroke, Infection, Inflammation

## Abstract

We aimed to determine if plasma levels of bacterial lipopolysaccharide (LPS) and lipoteichoic acid (LTA) are associated with different causes of stroke and correlate with C-reactive protein (CRP), LPS-binding protein (LBP), and the NIH stroke scale (NIHSS). Ischemic stroke (cardioembolic (CE), large artery atherosclerosis (LAA), small vessel occlusion (SVO)), intracerebral hemorrhage (ICH), transient ischemic attack (TIA) and control subjects were compared (n = 205). Plasma LPS, LTA, CRP, and LBP levels were quantified by ELISA. LPS and CRP levels were elevated in ischemic strokes (CE, LAA, SVO) and ICH compared to controls. LBP levels were elevated in ischemic strokes (CE, LAA) and ICH. LTA levels were increased in SVO stroke compared to TIA but not controls. LPS levels correlated with CRP and LBP levels in stroke and TIA. LPS, LBP and CRP levels positively correlated with the NIHSS and WBC count but negatively correlated with total cholesterol. Plasma LPS and LBP associate with major causes of ischemic stroke and with ICH, whereas LPS/LBP do not associate with TIAs. LTA only associated with SVO stroke. LPS positively correlated with CRP, LBP, and WBC but negatively correlated with cholesterol. Higher LPS levels were associated with worse stroke outcomes.

## Introduction

Stroke incidence increases with infection and inflammation prior to stroke^1^. C-reactive protein (CRP) levels after stroke correlate with stroke severity^2^; and, there is a whole genome immune response after stroke that differs for each stroke cause^3^. This response includes TNF, IL1, IL6 and other cytokines downstream of TLR4 and TLR2 pathways. Thus, we explored whether LPS (Lipopolysaccharide) or LTA (Lipoteichoic acid) levels are elevated in different causes of stroke and correlated with CRP levels since TLR4 is the receptor for Gram-negative bacterial LPS and TLR2 is the receptor for Gram-positive bacterial LTA, respectively.

LPS is increased in acute stroke and associated with poor short term outcome and long term mortality^4,5^. However, LPS levels were measured with the limulus lysate enzymatic assay which detects total LPS activity without identifying LPS molecules^6^. To solve this problem, we used a LPS specific ELISA for human plasma to quantify LPS, combined with an LPS binding protein (LBP) ELISA. LBP measurements, unlike LPS, are not subject to contamination.

Thus, this study assessed plasma levels of LPS, LBP, LTA, and CRP in patients with different causes of ischemic stroke, intracerebral hemorrhage (ICH) and transient ischemic attacks (TIAs) compared to controls. We hypothesize that levels of LPS and LTA, the inflammatory molecules from Gram-negative bacteria and Gram-positive bacteria, respectively, might change in some types of stroke and the LPS and LTA levels might correlate with LBP or CRP levels since LBP and CRP are acute phase proteins whose plasma concentrations change in response to inflammation.

## Methods

### Subject recruitment

Subjects with ischemic stroke (cardioembolic (CE, n = 33), large artery atherosclerosis (LAA, n = 42), small-vessel/lacunar (SVO, n = 41), intracerebral hemorrhage (ICH, n = 36), transient ischemic attacks (TIAs, n = 31), and controls (n = 22) were recruited at the University of California, Davis. The study was approved by the UC Davis Institutional Review Board and adhered to all federal and state regulations related to the protection of human research subjects, including the Common Rule, the principles of the Belmont Report, and institutional policies and procedures. Written informed consent for participation was obtained from all participants or their proxy. Ischemic stroke, ICH, and TIA subjects were recruited within 72 h of symptom onset. Exclusion criteria for all subjects were cancer, recent infection (< 4 weeks) or chronic infection including HIV.

Ischemic stroke, ICH, and TIA diagnoses were determined by two board-certified vascular neurologists. NIHSS, WBC count, lipid panel ((triglyceride (TG), total cholesterol (TC), high-density lipoprotein cholesterol (HDL-C), and low-density lipoprotein cholesterol (LDL-C)) were measured at admission. Non-HDL-C levels were calculated using TC levels subtracted from HDL-C levels. All TIA patients had their neurological symptoms resolve within 24 h and their brain MRIs were normal including DWI-MRI. Cause of ischemic stroke was determined using TOAST criteria from medical history, blood tests, Doppler and vascular angiography, cardiac investigations, and brain imaging. CE stroke required at least 1 source of cardiac embolus to be identified as well as the exclusion of LAA or SVO causes of stroke. CE sources included chronic atrial fibrillation, acute myocardial infarction, prosthetic valve, and cardiomyopathy. LAA stroke required > 50% stenosis of ipsilateral extracranial or major intracranial artery (middle cerebral artery, posterior cerebral artery or basilar artery) presumed to be due to atherosclerosis, and exclusion of CE or SVO causes of stroke. SVO strokes were defined as subcortical infarction less than 15 mm in longest diameter on brain imaging. Cryptogenic strokes (no cause, or more than one cause) or strokes of other determined causes such as arterial dissection, vasculitis or hypercoagulable states were excluded from this study. Intracerebral Hemorrhage (ICH) was confirmed by CT brain scan or gradient echo MRI. Patients with subarachnoid hemorrhage and hemorrhagic infarction were excluded. Controls were 22 healthy subjects similar in age, gender, and risk factors to stroke subjects. They had no history of stroke, cardiovascular disease or hematological disease, and had no infection within four weeks manifested either by clinical symptoms or temperature > 100° F.

Plasma samples were obtained on admission within 72 h of a stroke or TIA from each subject via venipuncture. Samples were collected in endotoxin free K_2_ EDTA 10 mL tubes (Becton Dickinson). Each sample was centrifuged at 1200×*g* for 10 min, plasma aliquoted and stored at − 80 °C. Extreme care was taken to keep all samples sterile and endotoxin/LPS free, and all processing was performed using sterile, LPS free reagents and glass/plastic ware.

### Measurement of LPS, LTA, LBP, and CRP

Sandwich ELISA kits were used to quantify LPS, LTA, LBP, and CRP. A standard curve was generated from known amounts of LPS, LTA, LBP, and CRP, and this was used to derive the values from patient plasma samples. Duplicate samples of plasma at different dilutions (1:100 dilution for LTA, 1:1000 dilution for LPS and LBP, and 1:4000 dilution for CRP) were measured according to protocol and the average used for statistical analyses. Human LPS ELISA kits (MBS266722, MyBioSource, Inc., San Diego, CA, USA), LTA ELISA kits (MBS772314, MyBioSource, Inc., San Diego, CA, USA), LBP ELISA kits (HK315, Hycult Biotech Inc. Wayne, USA), and CRP ELISA kits (KHA0031, Invitrogen, Carlsbad, CA, USA) were used to measure plasma levels. The monoclonal antibody against LPS used in the ELISA kits was raised to LPS from *E. coli* O111:B4, though it is unknown whether it cross-reacts to LPS from other Gram-negative bacteria.

### Statistics

A one-way ANOVA was performed with a Fisher LSD post hoc test to evaluate the differences of continuous variables among the groups if they passed normality test (age and LBP). For those continuous variables which failed normality test, Kruskal–Wallis test was employed (NIHSS, LPS, LTA, and CRP) followed by Dunn’s multiple comparison Post-hoc test. Chi-square test was used to evaluate the differences in sex, vascular risk factors and underlying diseases for different type of strokes among all groups followed by Fisher Exact test to evaluate the differences between stroke/TIA and controls. Pearson Correlation analysis was used to determine the correlations between the levels of the different analytes. Age was expressed as Mean ± standard error of the mean (SE) and other continuous variables were expressed as Median (IQR). A *p* value ≤ 0.05 was considered statistically significant. SigmaStat 2.03 software was used (San Jose, USA).

## Results

There were no significant differences in age, sex, hypertension, diabetes, and hyperlipidemia among the groups (Table 1). The incidences of atrial fibrillation and coronary artery disease were higher in some stroke groups compared to control (Table 1). As expected, the NIHSS on admission was significantly lower in the TIA group than stroke (*p* < 0.001) (Table 1). By 24 h the NIHSS was 0 in all TIA subjects who had normal brain MRI scans including DWI-MRI.

**Table 1:** Demographic variables in different type of stroke, TIA, and healthy controls.

	CE (n = 33)	LAA (n = 42)	SVO (n = 41)	ICH (n = 36)	TIA (n = 31)	Control (n = 22)	*p* value
Sex male n (%)	28 (85)	30 (71)	28 (68)	27 (75)	19 (61)	14 (64)	0.232
Age: years ± SE	65.2 ± 2.0	61.6 ± 1.4	65.3 ± 1.1	62.5 ± 2.4	65.1 ± 2.1	63.2 ± 2.0	0.496
Hypertension n (%)	27 (81.8)	35 (83.3)	34 (82.9)	31 (86.1)	24 (77.4)	12 (54.6)	0.069
Diabetes n (%)	9 (27.3)	18 (42.9)	23 (56.1)	11 (30.6)	10 (32.3)	7 (31.8)	0.095
Hyperlipidemia n (%)	16 (48.5)	22 (52.4)	25 (61.0)	11 (30.6)	20 (64.5)	9 (40.9)	0.054
AF (%)	18 (54.5)	2 (4.8)	5 (12.2)	3 (8.3)	3 (9.7)	0 (0)	< 0.001*
CAD (%)	10 (30.3)	10 (23.8)	3 (7.3)	6 (16.7)	7 (22.6)	0 (0)	0.023**
CHF (%)	2 (6.1)	1 (2.4)	1 (2.4)	1 (2.8)	4 (12.9)	0 (0)	0.181
Dementia n (%)	1 (3.0)	0 (0)	0 (0)	1 (2.8)	0 (0)	0 (0)	0.551
Parkinson’s disease n (%)	1 (3.0)	0 (0)	0 (0)	0 (0)	0 (0)	0 (0)	0.388
NIHSS: median (IQR)	5 (1–10.25)	3 (1–5)	4 (2–7)	4 (1–7)	0 (0–1)	–	< 0.001

*CE* cardioembolic, *LAA* large artery atherosclerosis, *SVO* small-vessel occlusion, *TIA* transient ischemic attack, *AF* atrial fibrillation, *CAD* coronary artery disease, *CHF* congestive heart failure, *SE* standard error of the mean, *NIHSS* National Institutes of Health Stroke Scale, *IQR* Interquartile range.

*p value for chi-square of total 6 groups. CE to control: *p* < 0.001; LAA to control: *p* = 0.542; SVO to control: *p* = 0.153; ICH to control: *p* = 0.281; TIA to control: *p* = 0.258.

**p value for chi square of total 6 groups. CE to control: *p* = 0.004; LAA to control: *p* = 0.012; SVO to control: *p* = 0.546; ICH to control: *p* = 0.073; TIA to control: *p* = 0.033.

**Figure 1:**
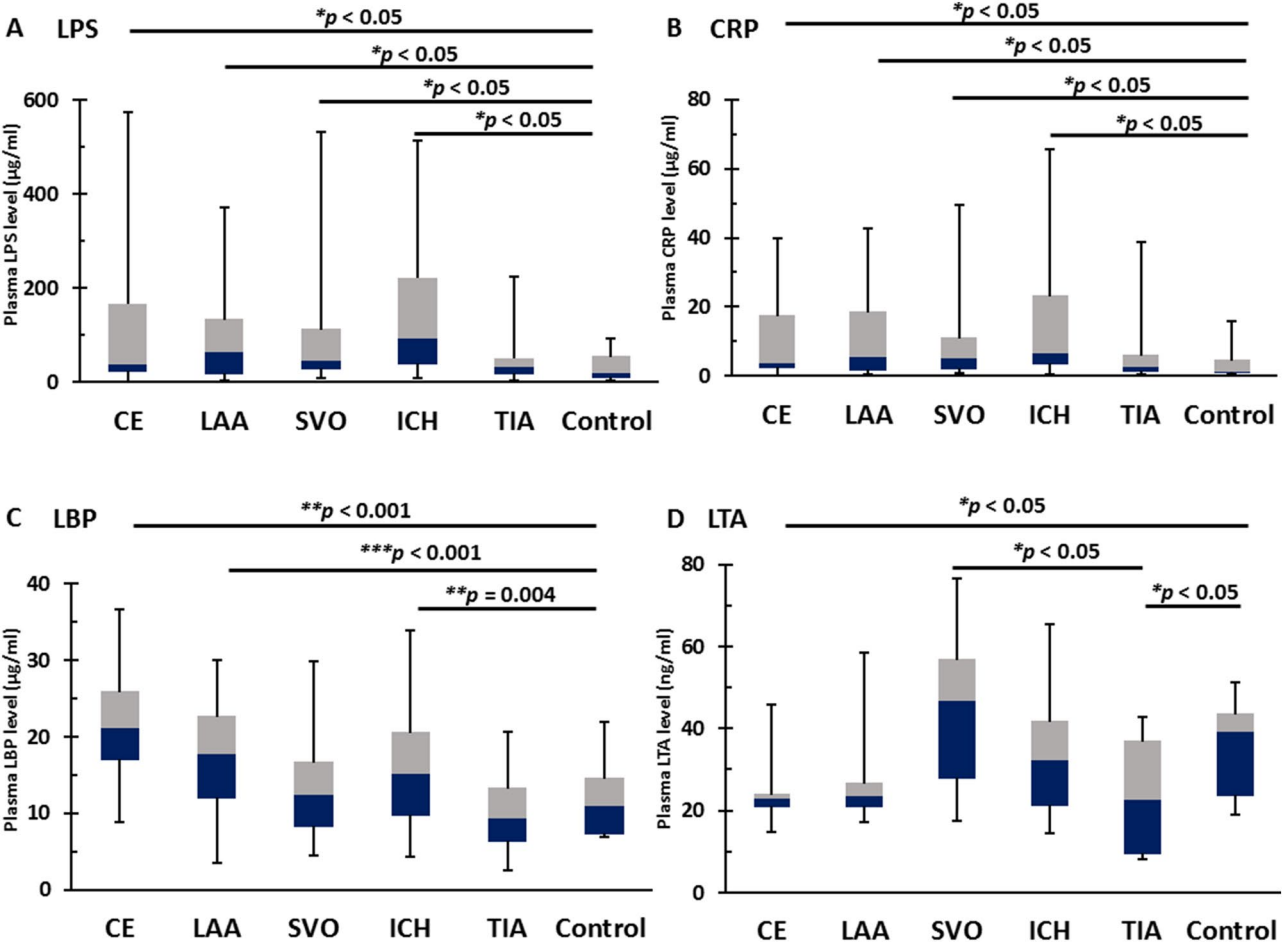
ELISA for LPS, LBP, CRP, and LTA for different causes of stroke and TIA vs controls. (**A**) The plasma levels of LPS, a component of the cell wall of all Gram-negative bacteria, were significantly higher in patients with intracerebral hemorrhage (ICH), CE stroke, LAA stroke, and SVO stroke compared to age-, sex- and vascular risk factor-matched healthy controls. The difference between LPS levels in TIA and healthy control was not significant. (**B**) The plasma levels of CRP, the classical acute-phase protein, were significantly higher in ICH, CE stroke, LAA stroke, and SVO stroke than healthy controls. The difference of CRP levels between TIA and healthy controls was not significantly different. (**C**) The plasma levels of LBP, an acute-phase protein that binds to LPS, were significantly higher in patients with CE stroke, LAA stroke, and ICH compared to healthy controls. The difference between LBP levels in SVO stroke and TIA were not significantly different from controls. (**D**) The plasma levels of LTA, a Gram-positive bacterial component, were significantly higher in SVO stroke compared to TIA. LTA levels were lower in CE stroke and TIA compared to controls. The difference of LTA levels between LAA stroke and ICH and healthy controls was not significant. *LPS* lipopolysaccharide, *LBP* lipopolysaccharide binding protein, *CRP* C-reactive protein, *LTA* lipoteichoic acid, *CE* cardioembolic, *LAA* large artery atherosclerosis, *SVO* small-vessel occlusion, *ICH* intracerebral hemorrhagic, *TIA* transient ischemic attack, *VRFs* vascular risk factors.

LPS levels in ICH (94.2 (38.9–223.7) µg/ml), CE strokes (37.6 (20.6–168.9) µg/ml), SVO stroke (45.7 (27.0–115.1) µg/ml), and LAA stroke (64.7 (16.0–136.2) µg/ml) but not TIA (31.9 (16.5–53.4) µg/ml) were significantly greater than controls (18.4 (8.6–56.8) µg/ml) (Fig. 1A). CRP levels in ICH (6.7 (3.3–23.6) µg/ml), CE strokes (3.6 (2.1–17.6) µg/ml), LAA stroke (5.4 (1.4–18.6) µg/ml), and SVO stroke (5.2 (1.8–11.2) µg/ml) but not TIA (2.7 (1.2–6.1) µg/ml) were significantly greater than controls (1.3 (0.8–4.7) µg/ml) (Fig. 1B).

LBP levels in CE stroke (21.1 (17.0–25.9) µg/ml), LAA strokes (17.8 (11.9–22.7) µg/ml), and ICH (15.1 (9.6–20.7) µg/ml) but not SVO stroke (12.4 (8.2–16.7) µg/ml) or TIA (9.4 (6.3–13.4) µg/ml) were significantly greater than controls (10.9 (7.3–14.7) µg/ml) (Fig. 1C). LTA levels in SVO stroke (46.7 (27.7–56.9) ng/ml) were not significantly greater than controls (39.1 (23.6–43.6) ng/ml) but were significantly greater than TIA (22.6 (9.4–37.2) ng/ml). LTA levels in CE strokes (23.0 (20.7–24.2) ng/ml) and TIA were significantly lower than controls (Fig. 1D). LTA levels in LAA stroke (23.5 (20.7–26.8) ng/ml) and ICH (32.3 (21.0–41.8) ng/ml) did not differ from controls and TIA (Fig. 1D).

**Figure 2:**
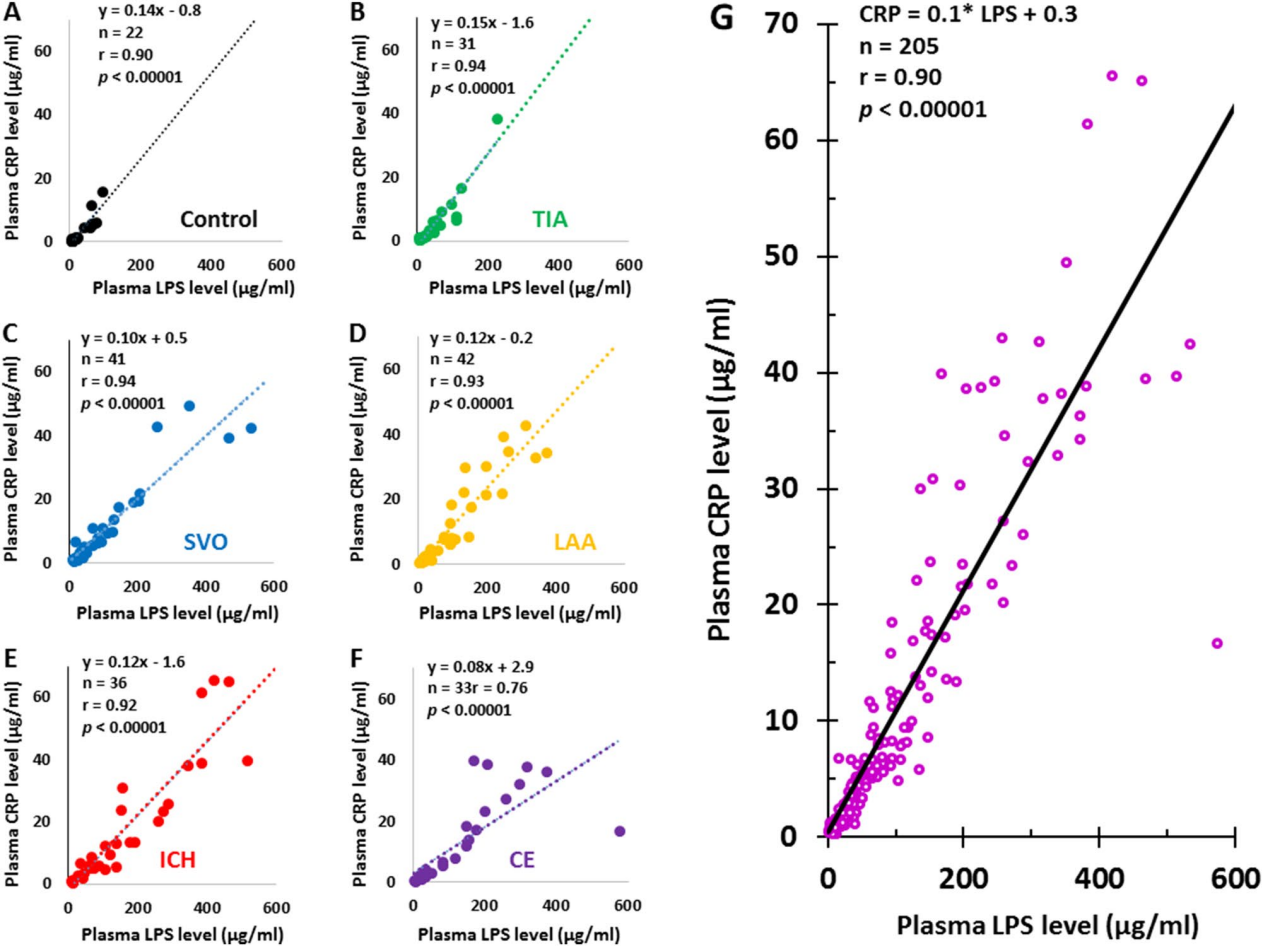
Pearson Correlation Analysis for plasma LPS levels and plasma CRP levels in different causes of stroke, TIA, and controls. The plasma LPS levels correlated with plasma CRP levels in controls (**A**), TIA (**B**), SVO stroke (**C**), LAA stroke (**D**), ICH (**E**), CE stroke (**F**) and for all subjects (**G**). Note that the intercepts of LPS trendlines on Y axis (CRP levels) in control, TIA, SVO stroke, LAA stroke, ICH stroke and CE strokes were − 0.8, − 1.6, 0.5, − 0.2, − 1.6 and 2.9 µg/ml, respectively (**A**–**F**). The intercept of LPS trendline on Y axis (CRP levels) for all 205 subjects was + 0.3 as indicated in (**G**). *LPS* lipopolysaccharide, *CRP* C-reactive protein, *CE* cardioembolic, *LAA* large artery atherosclerosis, *SVO* small-vessel occlusion, *ICH* intracerebral hemorrhagic, *TIA* transient ischemic attack.

**Figure 3:**
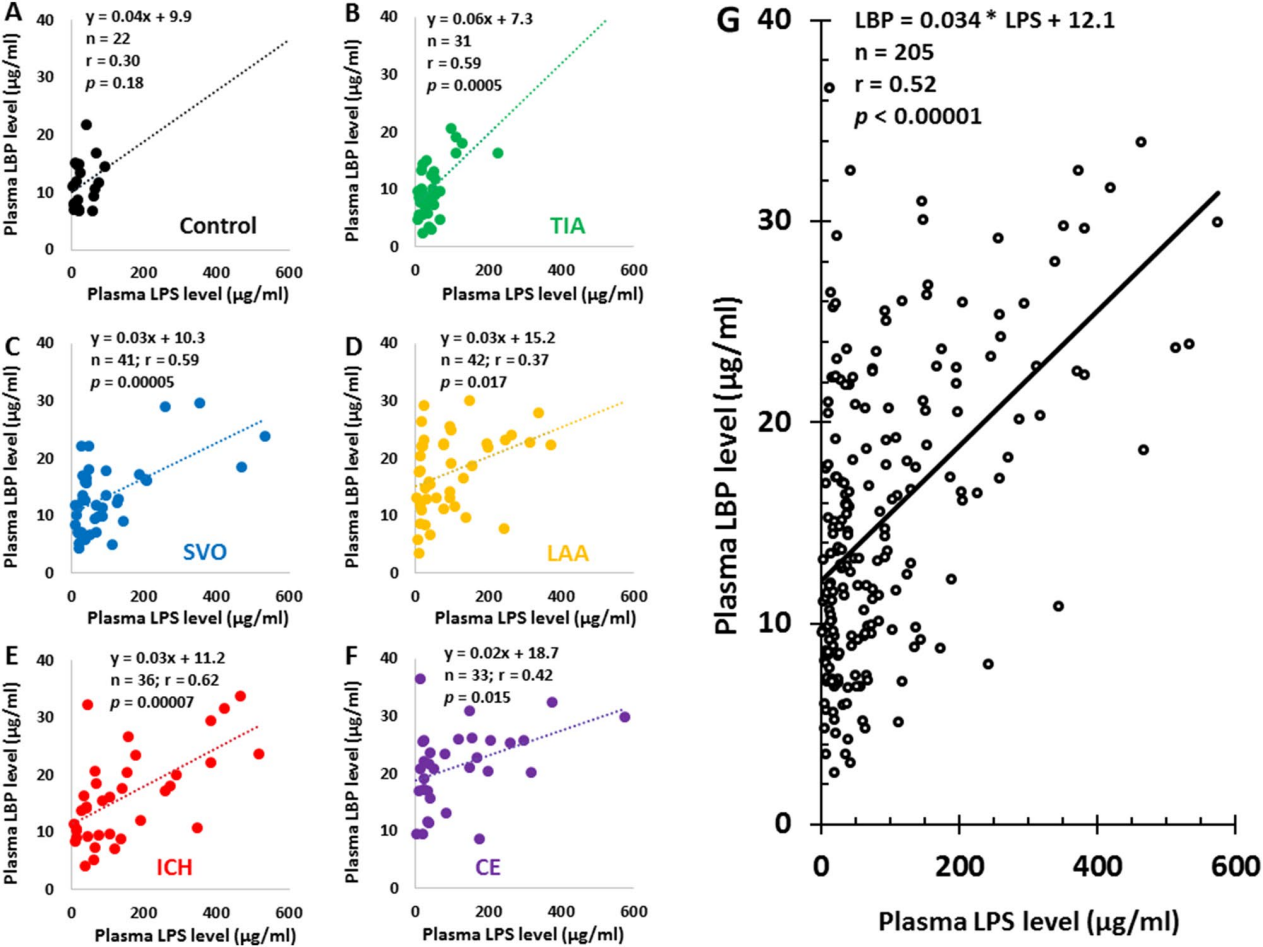
Pearson Correlation Analysis for plasma LPS levels and plasma LBP levels in different causes of stroke, TIA, and controls. The plasma LPS levels correlated with plasma LBP levels in TIA patients (**B**), SVO stroke (**C**), LAA stroke (**D**), ICH (**E**), CE stroke (**F**) and in all subjects (**G**) though not in controls (**A**). Note that the intercepts of LPS trendlines on Y axis (LBP levels) in control, TIA, SVO stroke, LAA stroke, ICH stroke and CE strokes were 9.9, 7.3, 10.3, 15.2, 11.2, and 18.7 µg/ml, respectively (**A**–**F**). The intercept of LPS trendline on Y axis (LBP levels) for all 205 subjects was 12.1 as indicated in (**G**). *LPS* lipopolysaccharide, *LBP* lipopolysaccharide binding protein, *CE* cardioembolic, *LAA* large artery atherosclerosis, *SVO* small-vessel occlusion, *ICH* intracerebral hemorrhagic, *TIA* transient ischemic attack.

Pearson Correlation analyses showed LPS levels correlated with CRP levels in controls (Fig. 2A), TIA (Fig. 2B), SVO stroke (Fig. 2C), LAA stroke (Fig. 2D), ICH (Fig. 2E), CE stroke (Fig. 2F), and all 205 subjects (Fig. 2G, r = 0.90, and *p* < 0.00001). The intercept for 0 LPS on the CRP axis was near zero (CRP, 0.3 µg/ml) (Fig. 2). Although LPS levels did not correlate with LBP levels in controls (Fig. 3A), LPS levels correlated with LBP in TIA (Fig. 3B), SVO stroke (Fig. 3C), LAA stroke (Fig. 3D), ICH (Fig. 3E), CE stroke (Fig. 3F), and all 205 subjects (Fig. 3G). The intercept for 0 LPS on the LBP axis was 12.1 µg/ml (Fig. 3G). LTA did not correlate with CRP levels in controls (Supplementary Fig. S1A), TIA (Supplementary Fig. S1B), SVO stroke (Supplementary Fig. S1C), LAA stroke (Supplementary Fig. S1D), ICH (Supplementary Fig. S1E), CE stroke (Supplementary Fig. S1F), and in all 205 subjects (Supplementary Fig. S1G).

**Figure 4:**
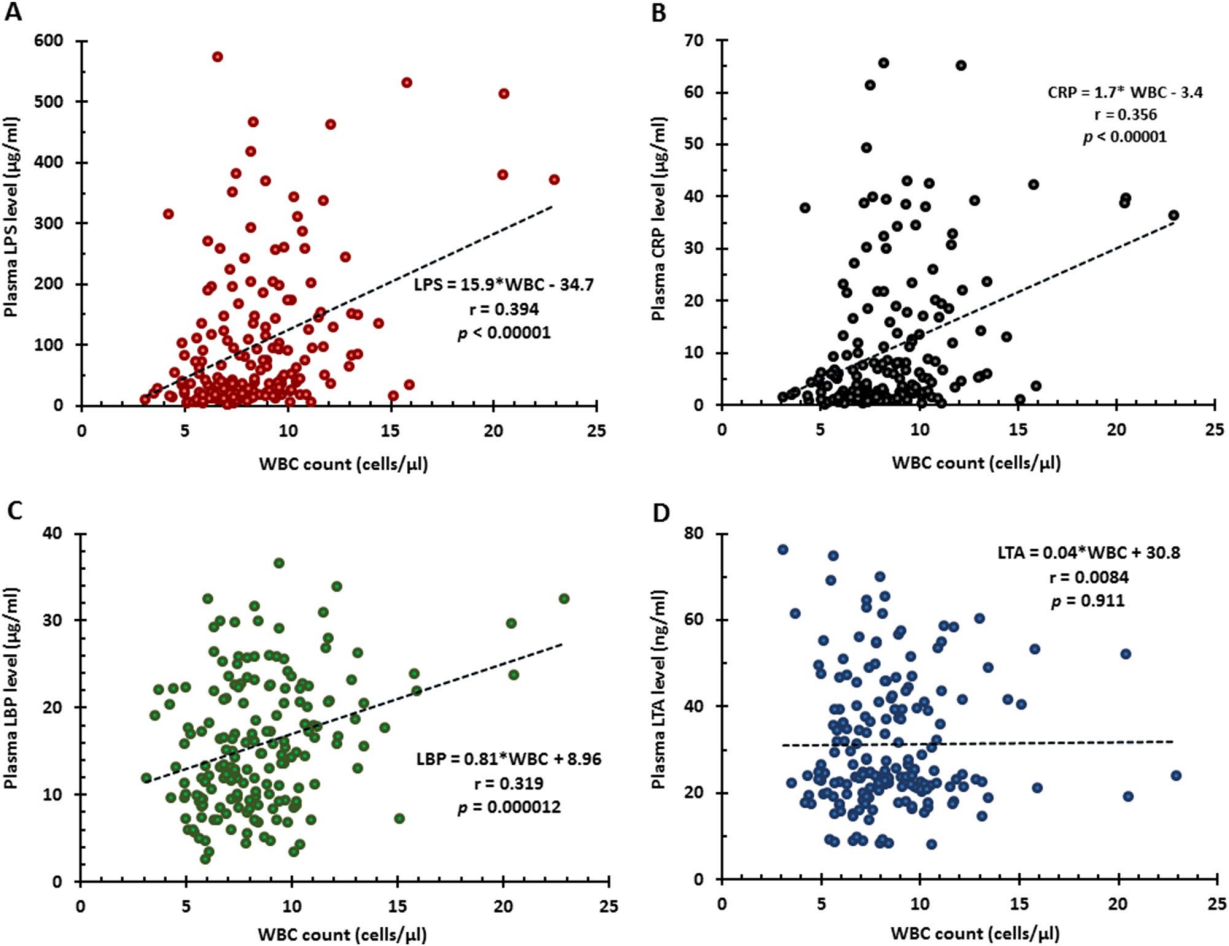
Pearson Correlation Analysis for WBC and levels of LPS, LBP, CRP, and LTA. WBC correlated with plasma LPS levels (**A**), CRP levels (**B**), LBP levels (**C**) but not with LTA levels (**D**). *WBC* white blood cell, *LPS* lipopolysaccharide, *LBP* lipopolysaccharide binding protein, *CRP* C-reactive protein, *LTA* lipoteichoic acid.

The levels of LPS (Fig. 4A), CRP (Fig. 4B), LBP (Fig. 4C) but not LTA (Fig. 4D) positively correlated with WBC counts. Similarly, the levels of LPS (Supplementary Fig. 2A), CRP (Supplementary Fig. S2B), LBP (Supplementary Fig. S2C) but not LTA (Supplementary Fig. S2D) positively correlated with percentage of neutrophils. In contrast to neutrophils, lymphocytes had negative correlations with the levels of LPS (Supplementary Fig. S3A), CRP (Supplementary Fig. S3B), LBP (Supplementary Fig. S3C) but not LTA (Supplementary Fig. S3D).

**Figure 5:**
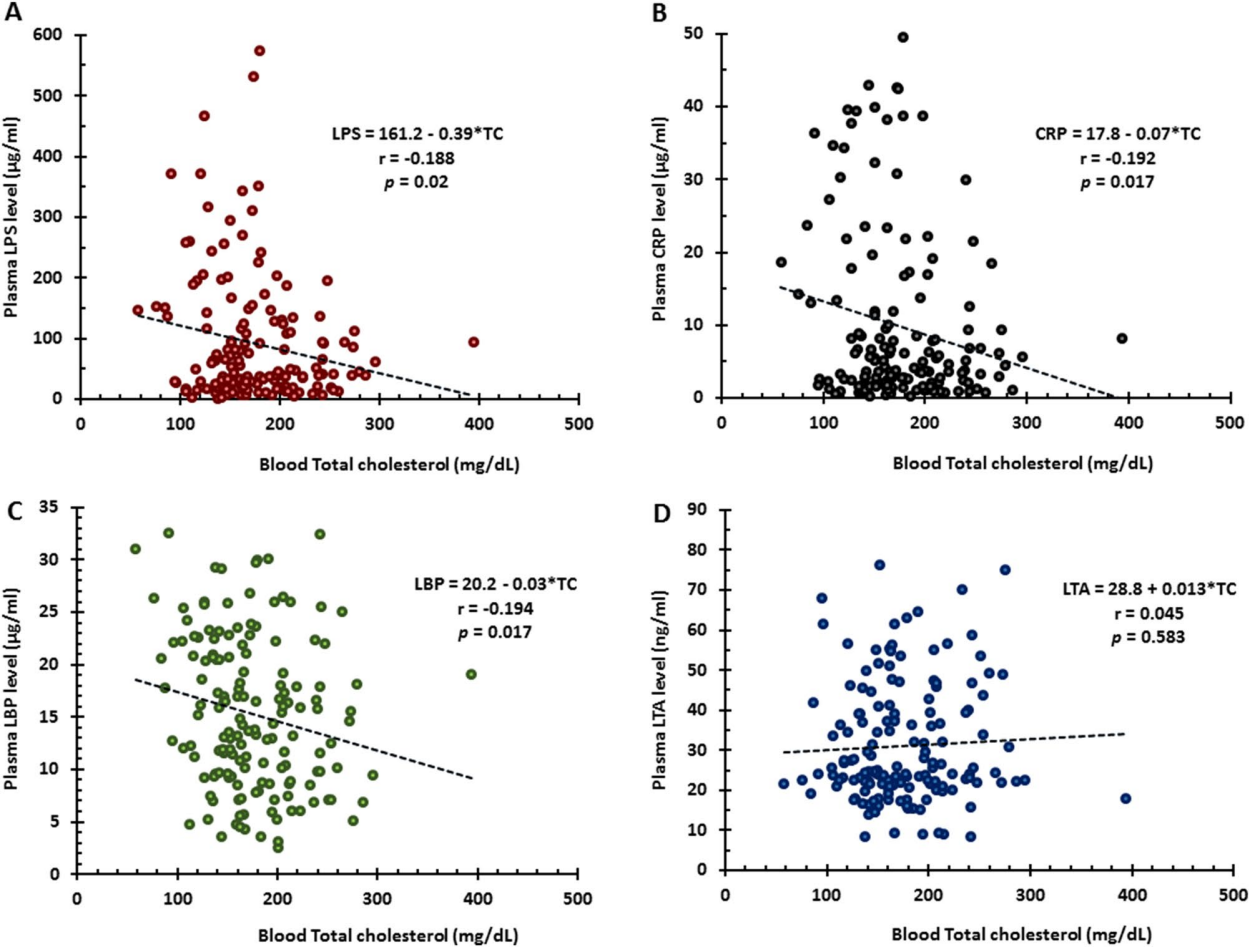
Pearson Correlation Analysis for total cholesterol and levels of LPS, LBP, CRP, and LTA. Total cholesterol negatively correlated with plasma LPS levels (**A**), CRP levels (**B**), LBP levels (**C**) but not with LTA levels (**D**). *TC* total cholesterol, *LPS* lipopolysaccharide, *LBP* lipopolysaccharide binding protein, *CRP* C-reactive protein, *LTA* lipoteichoic acid.

**Figure 6:**
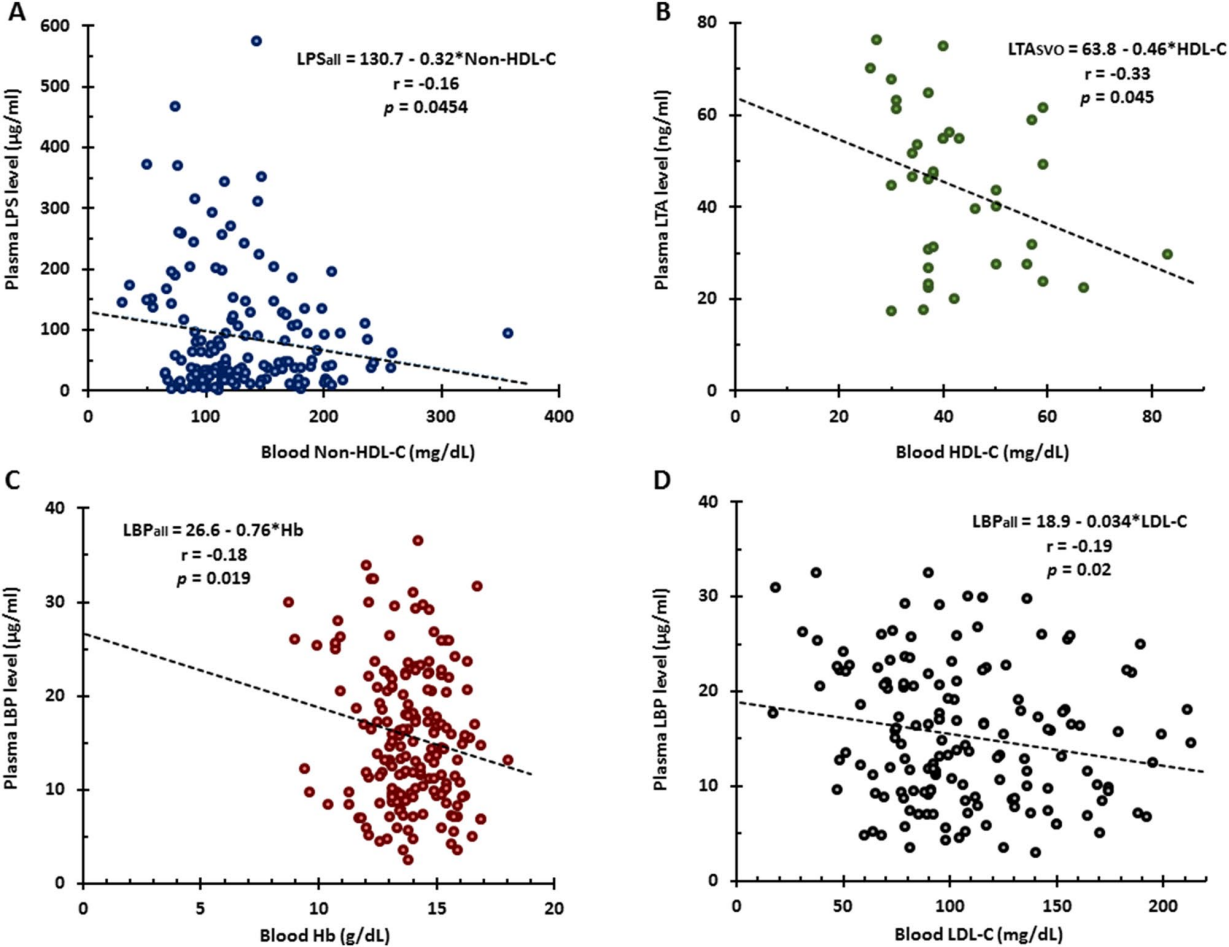
Pearson Correlation Analysis for Non-HDL-C and LPS levels, HDL-C and LTA levels, as well as hemoglobin and LDL-C and LBP levels. Non-HDL-C negatively correlated with plasma LPS levels (**A**). HDL-C negatively correlated with plasma LTA levels (**B**). Hb (**C**) and LDL-C (**D**) negatively correlated with plasma LBP. *Hb* hemoglobin, *LDL-C* low density lipoprotein-cholesterol, *HDL-C* high density lipoprotein-cholesterol, *Non HDL-C* non high density lipoprotein-cholesterol, *LPS* lipopolysaccharide, *LBP* lipopolysaccharide binding protein, *LTA* Lipoteichoic acid.

**Figure 7:**
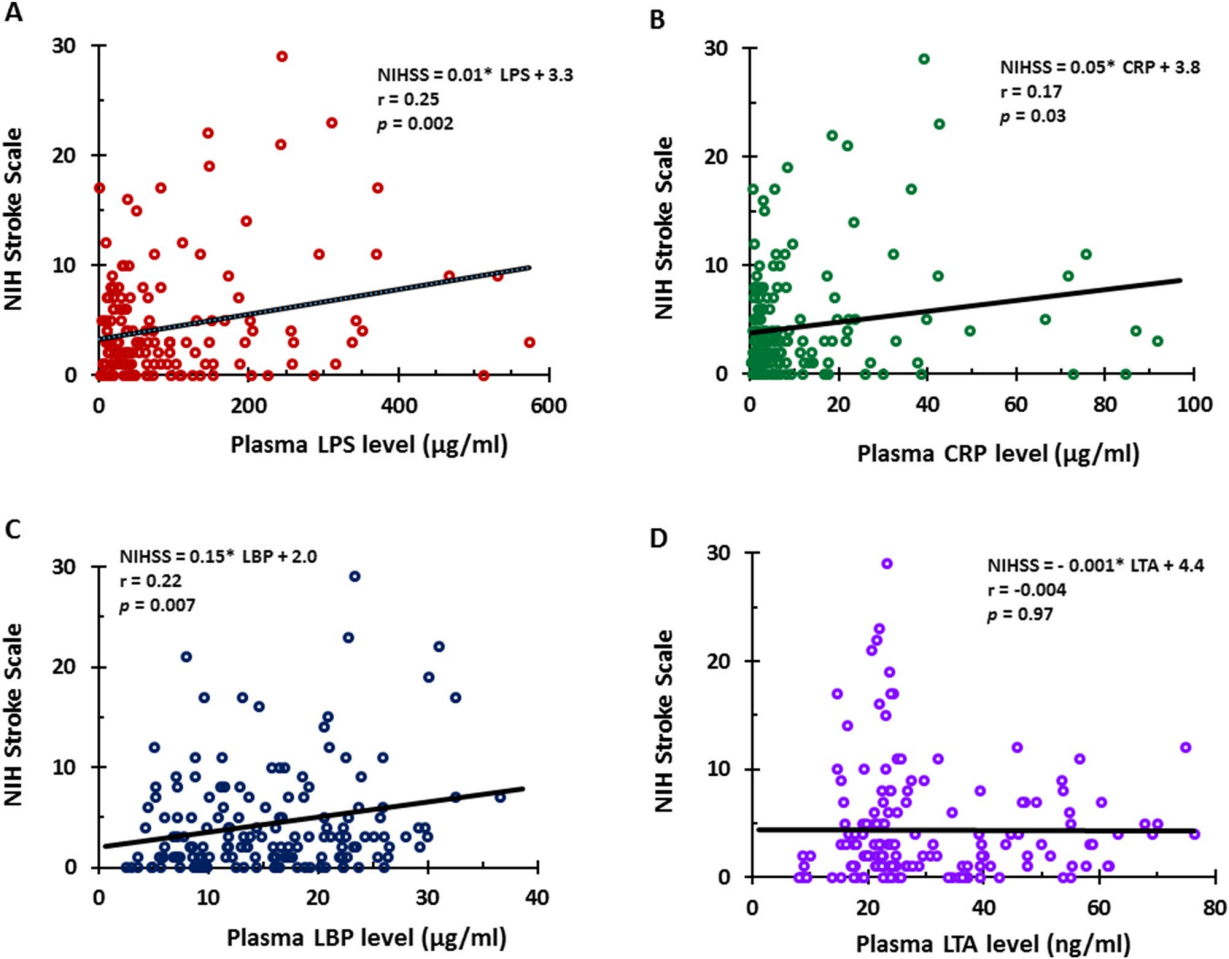
Pearson Correlation Analysis for NIHSS and levels of LPS, LBP, CRP, and LTA from TIA and different causes of stroke. NIHSS correlated with plasma LPS levels (**A**), CRP levels (**B**), LBP levels (**C**) but not with LTA levels (**D**). *NIHSS* NIH stroke scale, *LPS* lipopolysaccharide, *LBP* lipopolysaccharide binding protein, *CRP* C-reactive protein, *LTA* lipoteichoic acid.

The levels of LPS (Fig. 5A), CRP (Fig. 5B), LBP (Fig. 5C) but not LTA (Fig. 5D) negatively correlated with total cholesterol. Furthermore, LPS levels negatively correlated with Non-HDL-C (Fig. 6A) but not with HDL-C (not shown), LDL-C (not shown) and triglycerides (not shown). LTA levels negatively correlated with HDL-C only in SVO (Fig. 6B) stroke. In addition, hemoglobin (Fig. 6C) and LDL-C (Fig. 6D) negatively correlated with LBP. NIHSS values were significantly greater in CE, LAA, SVO strokes and ICH compared to TIA subjects (Supplementary Fig. S4). LPS levels (Fig. 7A), CRP levels (Fig. 7B), and LBP levels (Fig. 7C) positively correlated with the NIHSS. LTA levels did not correlate with the NIHSS (Fig. 7D).

## Discussion

This is the first study to evaluate plasma levels of both Gram-negative bacterial LPS and Gram-positive bacterial LTA in patients with different causes of stroke compared to controls. LPS levels are elevated in ischemic stroke (CE, LAA, and SVO) and ICH compared to controls. Since there might be some concern about LPS contamination, these findings were supported by elevated LPS-binding protein (LBP) levels in ischemic stroke (CE and LAA) and ICH compared to controls. In contrast, LTA levels only increased in SVO stroke compared to TIA but not controls. LPS levels correlate with CRP and LBP for all causes of stroke. LPS, LBP and CRP levels correlate with NIHSS. Since the half-life of LPS/LTA is short, we postulate that LPS and LTA in plasma are continually derived from the gut and other body organs like the gums and skin.

LPS levels have been associated with several risk factors for stroke and myocardial infarction including diabetes, hypertension, and high fat diet^7^. Since the risk factors in stroke compared to control subjects were not significantly different in our study, the elevated LPS levels associated with stroke suggests that LPS promotes inflammation that begins either prior to a stroke and/or following a stroke. Previous studies have demonstrated that LPS levels following human ischemic stroke correlate with worse short-term outcomes and mortality at 90 days^4,5^. This is consistent with LPS and LBP levels correlating with the NIHSS in the current study.

LPS may play different roles in different causes of stroke. LPS and LBP levels correlate with atherosclerosis in LAA stroke and with carotid intima-medial thickness (CIMT) in humans^8^ and experimentally LPS promotes atherosclerosis^8^. Indeed, LPS is found in carotid atherosclerotic plaque and blood LPS levels are higher in patients with carotid plaques compared to those without plaques^9^. LPS induces dysfunction of serum lipids that then accumulate on the aortic walls to form atherosclerotic plaques in ApoE−/− mice^10^, which might provide some insight into why LPS levels are higher in LAA patients.

Though there are no previous studies of LPS in cardioembolic stroke per se, a recent study did find that atrial fibrillation was associated with higher LPS levels, and that LPS levels were predictive of atrial fibrillation patients who developed MACE (Major Adverse Cardiovascular Events) that included a composite of myocardial infarction, ischemic stroke and TIAs, cardiac revascularization and cardiovascular death^11^. No data were given for stroke per se, however. Experimentally, LPS can promote atrial fibrillation and thus could contribute to human CE stroke. Finally, LPS can contribute to clot formation^12^ which plays a role in CE stroke as well as LAA and SVO stroke.

LBP and LPS have an intricate, interrelated biology. LBP binds LPS in blood. The LPS-LBP complex binds CD14 and triggers TLR4 on monocytes, macrophages, and microglia to bind LPS directly. This causes TLR4 internalization and downstream activation of MyD88-independent activation of NFκB and induces the production of IL1, IL6 and TNF^13^. Of the genes activated in blood following CE and LAA stroke, a number are down-stream of LPS^14^. In addition, LPS/TLR4 downstream genes were also activated following ICH stroke^15^. Our data are consistent with suggestions that there is a pro-inflammatory state either before a stroke and/or after a stroke which could contribute to causing a stroke and/or worsening outcome.

LPS induction of IL1 and particularly IL6 activate APRF/STAT3 which binds to the LBP gene promoter to increase the synthesis of LBP in hepatocytes and intestinal epithelial cells^16^. This likely explains the correlation of LPS levels with LBP levels with ischemic stroke, TIA, and ICH in the current study. That LPS drives LBP expression in stroke is supported by human studies where LPS infusion increases LBP levels^17^. However, when all of the subjects are considered, the LPS trendline for zero LPS intercepts the LBP axis at 12.1 µg/ml, which means LBP is expressed even when LPS is very low. Therefore, LBP is likely activated by and binds other molecules in addition to LPS. Indeed, LBP has been reported to recognize different ligands such as live bacteria^18^, glycolipids^19^, lipoproteins^20^ and peptidoglycan^21^.

CRP, an acute-phase protein, rapidly rises in serum in response to tissue injury and infection^22^. CRP is synthesized in hepatocytes in response to proinflammatory cytokines such as IL1, IL6 and IL17^23^, and mediates elimination of pathogens by recruiting complement and phagocytic cells^22^. CRP levels are increased in stroke and are associated with stroke incidence and outcomes. Since phosphorcholine (PC) is the principal ligand of CRP and PC is present in bacterial LPS^23^, we hypothesize that elevated LPS levels might directly drive CRP expression. Indeed, our results show CRP levels increase proportional to LPS levels in controls, TIA, ischemic stroke and ICH subjects, and the correlation coefficient is 0.90 (*p* < 0.00001) with the trendline for LPS vs CRP intercepting near zero on both axes. The proposal that LPS drives CRP expression is also consistent with LPS infusions in humans elevating plasma CRP^17^.

Lipoteichoic Acid (LTA) is found in the cell wall of almost all Gram-positive bacteria^24^, and LTA functions in parallel to LPS. LTA induces inflammatory responses by binding to TLR2 and inducing the innate immune system via different downstream regulators^25^. TLR2 signaling also has overlapping downstream pathways to TLR4. LTA binds TLR2 which signals to MyD88. However, TLR2-MyD88 activates the IRAK 1,2,4 complex. This then activates TRAF6 via a ubiquitin-linked pathway to induce downstream cytokines including IL1, IL1R, TNF and iNOS^26^. One of the most novel findings of the current study is that LTA levels are elevated only in SVO stroke, but not in LAA or CE stroke, or ICH, compared to TIA subjects. SVO stroke mainly involves penetrating arterioles, capillaries and venules, causes 25% of strokes and contributes to vascular dementia^27^. Though inflammation and infection have been implicated in SVO disease^27^, the causes have not been identified. The current study suggests that both LTA (TLR2) as well as LPS (TLR4) pathways are activated in SVO stroke and likely contribute to inflammation either before and/or after the SVO strokes. LTA activates bronchoalveolar coagulation via increases in the levels of thrombin-antithrombin complexes, d-dimer, and soluble tissue factor^28^ and induces similar changes in coagulation and fibrinolysis as LPS^28^. LTA activates the coagulation pathway in the lungs through TLR2-dependent pathway and the process is likely amplified by endogenous TLR4 ligands^29^. Of interest is a finding from human umbilical vein endothelia cells (ECs) that shows peptidoglycan rather than LTA induces EC ICAM-1 and VCAM-1 to facilitate the adhesion of monocytes, whereas both LTA and peptidoglycan significantly increase the expression of pro-coagulant Tissue Factor in ECs^30^. Further studies are required to evaluate any role for peptidoglycan in SVO or other causes of stroke.

Our data raise the interesting question of why LTA is only associated with SVO whereas LPS is associated with both SVO and LAA? Thus, we hypothesize that LPS/TLR4 is expressed in both small and large vessels whereas LTA/TLR2 may be expressed only in small vessels. In a previous study we have shown that LPS is localized in both small and large vessels of both Alzheimer’s Disease and normal control brains^31^. Future studies will need to determine if LTA/TLR2 is expressed exclusively in small vessels, and thus potentially explain its specific role in SVO but not other causes of stroke.

Notably, LTA levels did not correlate with CRP levels, in spite of the fact that Gram- positive infections have been shown to increase CRP^32^. This might be because of different CRP isoforms^32^, only one of which is detected by ELISA in this study. Alternatively, it is possible that Gram-positive bacteria induce CRP via different molecules than LTA, and these molecules are not increased with stroke since there probably are no live Gram-positive bacteria in blood following most strokes.

TIA subjects had LPS, LBP and CRP levels that were similar to controls and LTA levels in TIA patients were lower than control. The TIA subjects in these analyses were MRI DWI negative, and thus did not have minor strokes. About ~ 6.4% of TIAs go on to have a cardiovascular event including stroke^33^. Whether TIA subjects who go on to have strokes have higher LPS levels requires further study, particularly since we identified two types of TIA based upon gene expression in blood^34^.

LPS positively correlated with WBC counts and neutrophil percentage, which is consistent with an animal study showing LPS increases WBC and neutrophil counts^35^. Moreover, LPS challenge in humans also increases WBC/neutrophil counts associated with increased levels of CRP^36,37^ and LBP^38^. As mentioned earlier, CD14 acts as a co-receptor along with TLR4 to bind LPS in the presence of LBP. CD14 is a 55-kDa glycoprotein which exists in two forms: membrane-bound CD14 (mCD14) which is found on monocytes, macrophages, and neutrophils, and soluble CD14 (sCD14) which is present in plasma^39,40^. Both mCD14 and sCD14 mediate biological responses to LPS. It has been demonstrated that neutrophils respond to LPS/LBP complexes via CD14 to release TNFα^41^. Our study demonstrates a direct relationship between the levels of inflammatory markers of LPS, LBP and CRP and the percentage of neutrophils. Higher WBC counts and higher percentage of neutrophil without infection are likely a result of a LPS-induced proinflammatory state with elevated LBP and CRP as well. Given the critical role of neutrophils in mediating clot formation, this provides an additional reason for considering LPS as a risk factor for strokes.

An unexpected finding of the current study was that LPS levels negatively correlated with total cholesterol and non-HDL-C. Because cholesterol lipid has minimal solubility in water, free cholesterol levels in blood are exceedingly small. Instead, cholesterol is packaged within lipoproteins complexes which allow cholesterol to travel through blood via emulsification. Several types of lipoproteins exist in blood including chylomicrons, very-low-density lipoprotein (VLDL), intermediate-density lipoprotein, LDL, and HDL. Thus, cholesterol exists in blood in a free form and as cholesterol esters within lipoprotein particles. Our study demonstrates a significant inverse relationship between LPS levels and total cholesterol levels as well as non-HDL-C levels. Decreases in cholesterol during inflammatory diseases have been observed for more than a century in patients with bacterial and viral infections, critically ill patients, trauma patients, postoperative patients and patients with sepsis^42^. Thus, our findings are consistent with the previous literature showing that pro-inflammatory states, including the presence of LPS in our study, are associated with decreases of cholesterol in blood.

The mechanism by which LPS lowers cholesterol, however, is less clear. LPS is a large molecule consisting of a lipid and a polysaccharide. LPS can bind lipid transfer protein including cholesteryl ester transfer protein (CETP), lipoproteins and LBP. LPS markedly increases LBP expression and decreases hepatic CETP mRNA expression, plasma CETP concentration, plasma total cholesterol levels and non-HDL-C levels^43^, which might explain the direct relationship between LPS levels and LBP levels and inverse relationship between LPS levels and total cholesterol levels as well as non-HDL-C levels found in our study. Animal and human studies demonstrate that LPS decreases plasma CETP concentration^43^. Lipoproteins, particularly HDL^44,45^ and LDL^46^, bind and neutralize LPS and attenuate LPS induced biological responses in vivo and in vitro^47,48^. In fact, a large fraction of LPS enters the bloodstream by binding to HDL as a LPS-HDL complex^49^. Since HDL binds both cholesterol and LPS, plasma LPS might compete with cholesterol to bind HDL and cause an inverse relationship of LPS with total cholesterol and non-HDL-C found in our study. Mechanistic studies are required to assess this hypothesis.

Our study also demonstrated that LBP levels positively correlated with LPS but negatively correlated with LDL-C. As noted above, LPS increases LBP expression and decreases hepatic CETP mRNA expression, plasma CETP concentration, plasma total cholesterol levels and non-HDL-C levels in animal studies. CETP and LBP together with bactericidal/ permeability-increasing (BPI) and phospholipid transfer protein (PLTP) belong to members of a lipid transfer/LBP gene family^50–53^ which are produced in liver and participate in the innate immune response. Proteomic studies of human plasma demonstrate that LBP is present in the VLDL but not in LDL^54^. LBP is carried on lipoprotein and transfers LPS to lipoproteins in addition to CD14^40^. These studies suggest that both LBP and lipoproteins may be involved in LPS associated cholesterol changes in stroke patients. Since we did not measure lipoprotein levels, the relationship between LPS, LBP, lipoproteins and cholesterol in stroke patients remains to be elucidated.

Endothelial cells exposed to LPS show prothrombotic properties combined with enhanced Tissue Factor activity^55^. This may be partly associated with hemoglobin binding to LPS. Hb is present in high concentrations in red blood cells (RBCs). Hb has the capacity to bind LPS and enhance LPS biological activity^56,57^. Addition of Hb to cultured macrophages significantly enhanced LPS-dedicated cytokine production. Normally, LPS activity is regulated by LBP. However, Hb also binds LPS and forms a stable Hb-LPS complex which enhances the procoagulant activity of peripheral blood cells and tissue factor activity of endothelia^58^. Our current study demonstrates an inverse relationship between Hb concentrations and plasma LBP levels. Further study is required to determine whether Hb-LPS-LBP interactions are a risk factor for stroke.

The NIHSS correlated with LPS levels, CRP levels, and LBP levels. This finding would suggest that higher LPS levels produced a greater “pro-inflammatory” state that was associated with worse stroke outcomes. Indeed, higher LPS levels and higher CRP levels have previously been associated with increased morbidity and mortality following stroke^4,5^.

LPS has a short half-life of a few hours in plasma. The finding of increased LPS levels in stroke suggests there is a continuous release of LPS into blood. Recent evidence suggests that gut bacterial molecules or bacterial molecules from other sites like the gums and skin leak into the blood, and that stroke risk factors including hypertension, high fat diet, diabetes and others increase the permeability of gut and gum endothelial cells and thus contribute to inflammation caused by a “leaky gut” or inflamed gums^11^. LPS identified in this study was from *E. coli* O111:B4, which indicates a possible gut source. Our findings suggest that the gut may be leakier in stroke patients. Leakage of LPS from Gram-negative bacteria in the gut or gums to blood provides one model of how the gut or gum microbiome may affect stroke and other CNS diseases^59^. Stroke risk factors and stroke itself might lead to a “leaky gut” and increases of blood LPS via the autonomic nervous system, brain-gut endocrine interactions, and miRNA released from brain and brain-gut immune interactions. The finding that Gram-negative LPS is elevated in all causes of stroke including ICH, whereas Gram-positive LTA is elevated only in SVO stroke, suggests complex brain-gut-immune interactions that may be specific for molecules from specific classes of bacteria.

Limitations to the current studies include the fact that other TLR were not examined, so they have an uncertain role in stroke. The lower than control levels of LTA in CE and TIA subjects is of uncertain significance but could relate to factors not controlled for in our analyses including drugs given post-stroke. The study will need to be replicated in a larger cohort, and though age, hypertension, diabetes, and lipids were controlled for, future studies should consider additional variables like smoking, alcohol, BMI, and other vascular risk factors. Future studies will need to compare males and females and examine the effects of race and increasing age on LPS and LTA.

## Supplementary Information

Supplementary Legends.

Supplementary Figure S1.

Supplementary Figure S2.

Supplementary Figure S3.

Supplementary Figure S4.

## Data Availability

The corresponding author has access to all the data. The data will be made available upon written request.
