# Supplementary material for: Bacterial lipopolysaccharide is associated with stroke
Source: Sci Rep. 2021 Mar 22;11:6570. doi: 10.1038/s41598-021-86083-8 (PMC7985504; doi:10.1038/s41598-021-86083-8)
Supplement: Supplementary file 1 — Supplementary Legends. [file 41598_2021_86083_MOESM1_ESM.docx]

**Supplementary Fig. 1 Pearson Correlation Analysis for plasma LTA levels and plasma CRP levels in different causes of stroke, TIA, and controls.** The plasma LTA levels did not correlate with plasma CRP levels in controls (**A**), TIA patient **(B)**, SVO stroke **(C)**, LAA stroke **(D)**, ICH **(E)**, CE stroke **(F)** nor in all subjects **(G)**. Note that in control, TIA, and CE stroke, the r values were negative though the *p* values were not significant in any of the groups. Note that the intercepts of LTA trendlines on Y axis (CRP levels) in control, TIA, SVO stroke, LAA stroke, ICH stroke and CE strokes were 4.2, 6.1, 9.0, 8.3, 6.3, and 27.9 µg/ml, respectively (**A-F**). The intercept of LTA trendline on Y axis (CRP levels) for all 205 subjects was 8.5 as indicated in **G**. LTA = Lipoteichoic acid; CRP = C-reactive protein; CE = cardioembolic; LAA = large artery atherosclerosis; SVO = small-vessel occlusion; ICH = intracerebral hemorrhagic; TIA = transient ischemic attack.

**Supplementary Fig. 2 Pearson Correlation Analysis for percentage of neutrophil and the levels of LPS, LBP, CRP, and LTA.** Percentage of neutrophil positively correlated with plasma LPS levels (**A**), CRP levels (**B**), LBP levels (**C**) but not with LTA levels (**D**). LPS = lipopolysaccharide; LBP = lipopolysaccharide binding protein; CRP = C-reactive protein; LTA = Lipoteichoic acid.

**Supplementary Fig. 3 Pearson Correlation Analysis for percentage of lymphocyte and the levels of LPS, LBP, CRP, and LTA.** Percentage of lymphocyte negatively correlated with plasma LPS levels (**A**), CRP levels (**B**), LBP levels (**C**) but not with LTA levels (**D**). LPS = lipopolysaccharide; LBP = lipopolysaccharide binding protein; CRP = C-reactive protein; LTA = Lipoteichoic acid.

**Supplementary Fig. 4 NIH Stroke Scale in different causes of stroke vs TIA.** NIH Stroke Scale (NIHSS) was higher in patients with CE stroke, LAA stroke, SVO stroke, and ICH compared to TIA. CE = cardioembolic; LAA = large artery atherosclerosis; SVO = small-vessel occlusion; ICH = intracerebral hemorrhage; TIA = transient ischemic attack.
