# Supplementary figures and images for: Bacterial lipopolysaccharide is associated with stroke
Source: Sci Rep. 2021 Mar 22;11:6570. doi: 10.1038/s41598-021-86083-8 (PMC7985504; doi:10.1038/s41598-021-86083-8)

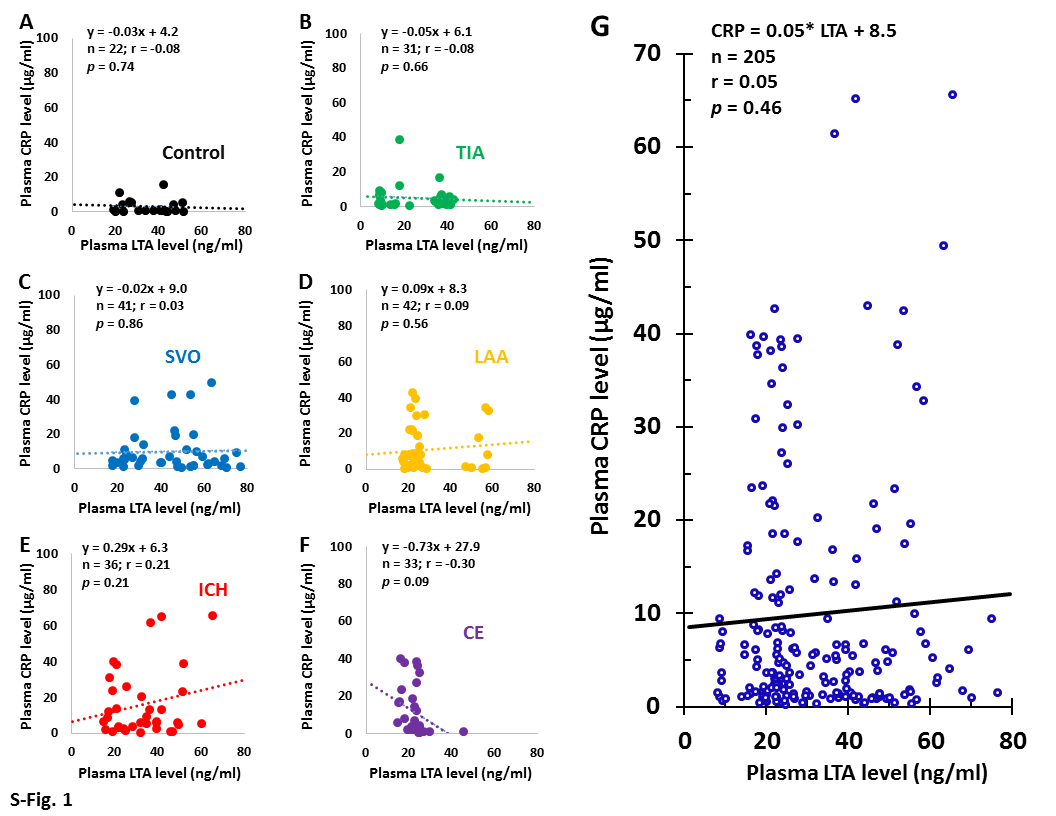

Supplement: Supplementary file 2 — Supplementary Figure S1. [file 41598_2021_86083_MOESM2_ESM.tif]

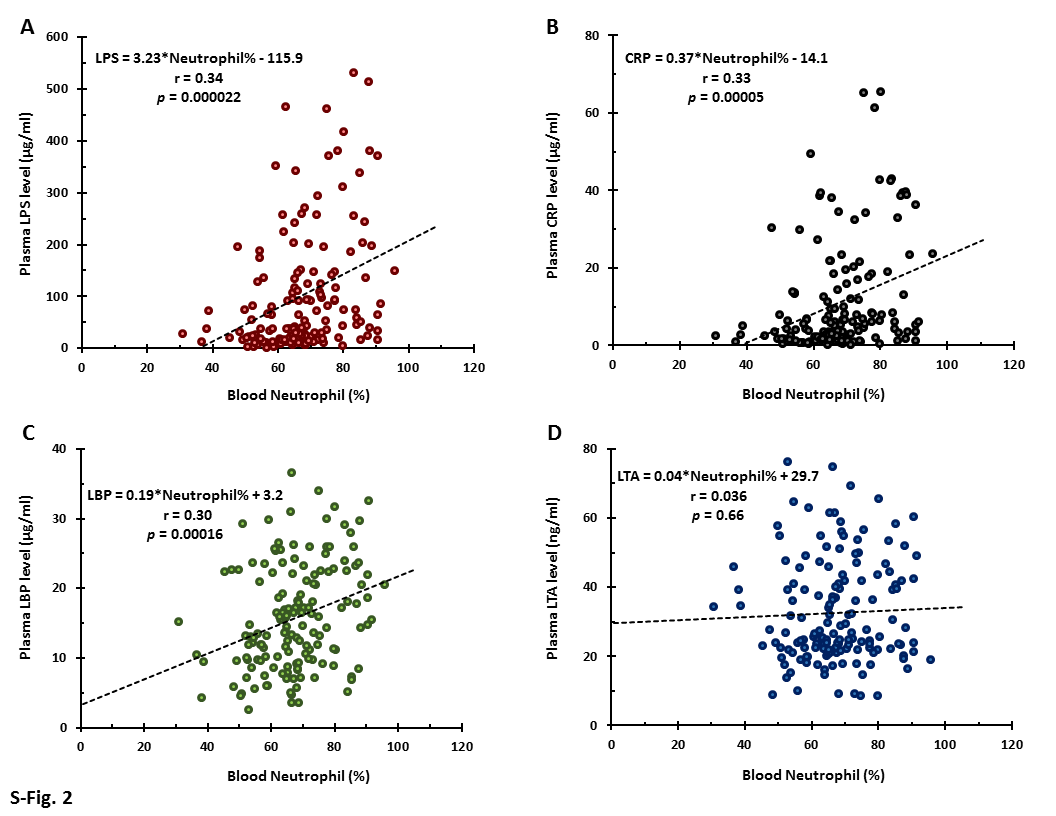

Supplement: Supplementary file 3 — Supplementary Figure S2. [file 41598_2021_86083_MOESM3_ESM.tif]

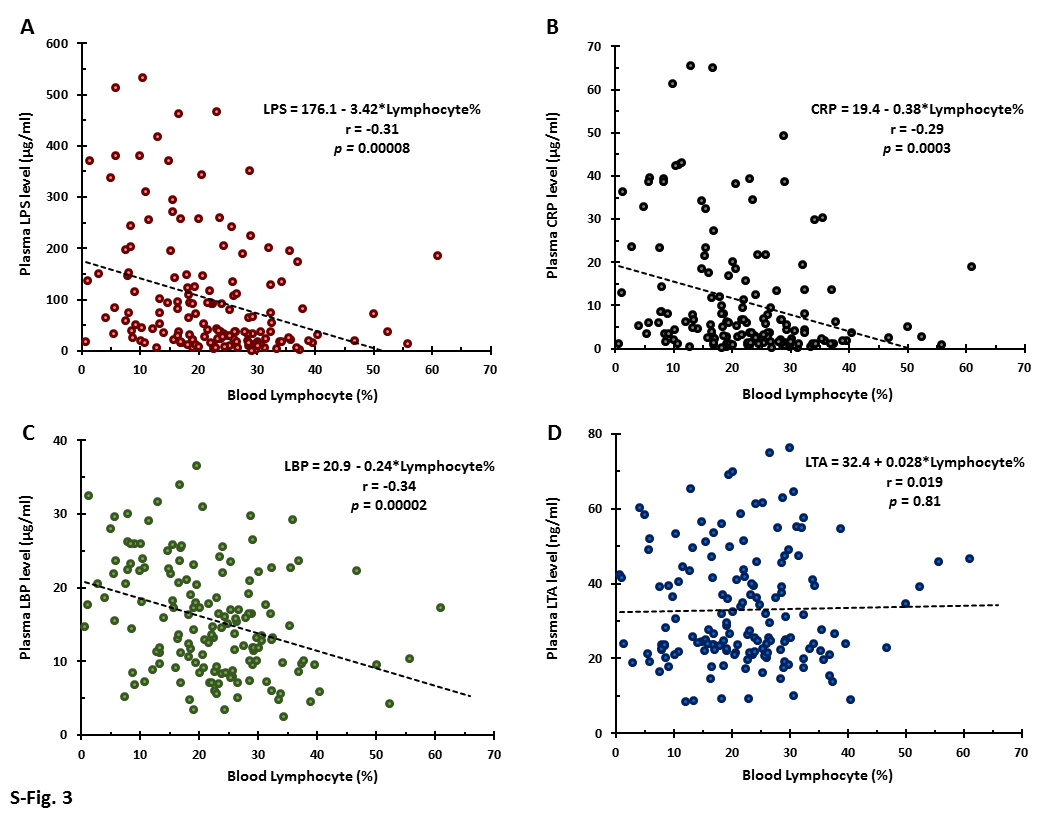

Supplement: Supplementary file 4 — Supplementary Figure S3. [file 41598_2021_86083_MOESM4_ESM.tif]

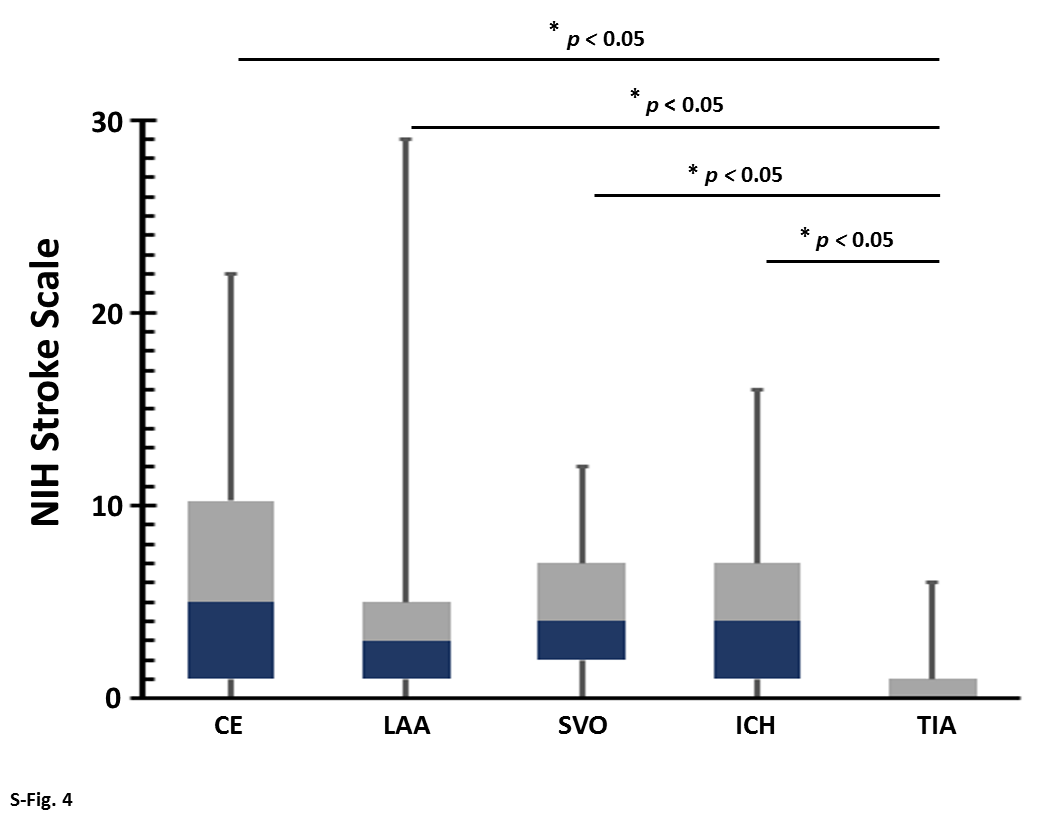

Supplement: Supplementary file 5 — Supplementary Figure S4. [file 41598_2021_86083_MOESM5_ESM.tif]
